# Role of cancer literacy in cancer screening behaviour among adults of Kaski district, Nepal

**DOI:** 10.1371/journal.pone.0254565

**Published:** 2021-07-13

**Authors:** Reecha Koirala, Nisha Gurung, Sarita Dhakal, Sulata Karki

**Affiliations:** 1 Department of Community Programs, Dhulikhel Hospital, Kathmandu University Hospital, Dhulikhel, Nepal; 2 Department of Community Medicine, Gandaki Medical College Teaching Hospital and Research Centre, Lekhnath, Gandaki Province, Nepal; 3 Kathmandu Model Research Foundation, Kathmandu, Nepal; Imperial College London, UNITED KINGDOM

## Abstract

Cancer Screening is a key approach to detect cancer at an early stage and help reduce cancer mortality globally. Inadequate Cancer Literacy may pose a barrier to patient engagement in getting screened for cancer. This study assessed Cancer Screening behavior and its association with Cancer Literacy and other factors among adults of Kaski district, Nepal. A cross-sectional study was carried out among 180 adults from March to August 2019, selected using a multi-stage random sampling method. Data on demographics, history of cancer, use of naturopathy, fatalism, family support, cancer literacy and cancer screening behaviour were collected using a semi-structured questionnaire, with the aid of face-to-face interviews. Cancer Literacy was measured using a cancer health literacy tool (CHLT-6), and Cancer Screening behaviour was assessed on the basis of the self reported information about having gone through any type of cancer screening in the past. Odds ratio (OR) with 95% Confidence Interval (CI) was calculated to determine the strength of association using Multivariate Logistic Regression analysis. Only 43.4% of the respondents had Cancer Literacy scores more than the median and only 11.7% had ever gone through any Cancer Screening test in the past. In this study, Cancer Screening behaviour was significantly associated with Cancer Literacy [OR = 1.43, 95% CI (1.01–2.02)]. Similarly, significant association was found between Cancer Screening behaviour and other exposure variables such as age [OR = 1.06, 95% CI (1.02–1.11)] and gender [OR = 0.06, 95% CI (0.01–0.35)]. This study showed low cancer screening and cancer literacy scores amongst the respondents. This suggests that to tackle the ever increasing burden of cancer and hence, to increase cancer screening, we need to focus on improving knowledge and awareness about cancer, as well as, on targeting efforts towards people’s understanding of basic health and cancer terminologies.

## Introduction

Cancer, at present, is a widely known non-communicable disease in the world, and Nepal is no exception to this health problem. In Nepal, the total number of deaths due to cancer had reached 186,000 in 2014, with more deaths among the females [1]. Approximately, 8,000 to 10,000 cases are identified annually, on the basis of hospitals’ data [2]. There are more males dying from lung and oral cavity cancers whereas more females dying from cervical and breast cancers in the context of Nepal [2]. The incidence of cancer in Nepal is estimated to reach 39.6% in Nepal in the year 2020 [3].

As per the 2018 national annual health report, a total of 13,997 cases were reported in the Out-patient Departments (OPDs) of the country, and among those, 27.6% cases were from Gandaki Province [4]. Within this province too, Kaski tops the list of districts being the most affected by cancer disease [5].

With the changing lifestyle and exposure to risk factors like tobacco smoking, excessive alcohol consumption, household solid fuel, physical inactivity and obesity, environmental pollution, and excessive use of pesticides in edibles, there has been a significant rise in the number of cancer patients in Nepal [4]. This shows that most of the cancers in the national context are preventable to some degree, where screening plays a major role.

Screening facilitates an early identification of undetected health conditions with the help of tests [6], and screening for cancer has been a key approach to reduce cancer mortality [7–11]. Screening for cancer gives an advantage to a person to find out about the disease at an earlier stage increasing chances of survival [7–11]. Screening for cancers has been recommended globally and nationally [12–15]. However, an individual’s response to screening can be influenced by their beliefs, attitudes, personal backgrounds, and access to care [8, 16, 17].

Closely aligned with cancer screening is cancer literacy [18, 19]. Cancer literacy is defined as all the knowledge a layperson needs to possess to understand the information and advice the health system has to offer regarding preventing, diagnosing and treating cancer [20]. In the context of Nepal, cancer literacy can be a critical issue since the information provided by the health workers may not be clear to the people receiving it, due to the existing low literacy rate, as well as fewer health workers per patient [21].

However, there is a dearth of scientific research studies focusing on this sector of cancer prevention and awareness, specifically cancer literacy and cancer screening behaviour, in general. Thus, we have attempted to assess cancer literacy, its relationship with cancer screening behaviour, if any, and identify other factors associated with the screening behaviour. We have particularly chosen Kaski district, since it has one of the highest numbers of cancer cases [5], as well as has more prospect of improvement since many cancer prevention pilot and full-fledged programs are being conducted in this region [4]. We believe this study generates some evidence with which the concerned governmental and non-governmental bodies can work to strengthen cancer awareness and prevention campaigns.

## Materials and methods

### Ethics statement

This study was approved by the Ethical Review Board of Nepal Health Research Council, Kathmandu, Nepal *(Reg*. *No*. *256/2019)*. Participants were explained about the study and its importance, and written informed consent was obtained from the willing participants before enrolling them in the survey. If they were unable to write, their fingerprints were taken to denote their consent. They were informed that their participation would be voluntary, their responses will be kept strictly confidential and be only used for the purpose of this study. Their anonymity was maintained using strict coding measures (i.e. coding names of the respondents in numbers).

### Study setting, design and population

We conducted a cross-sectional research in the randomly selected wards of rural and urban municipalities of the Kaski district of Gandaki Province from March to August 2019. A ward is the smallest local administrative unit in Nepal and there are a total of 6684 wards, which make up to 744 local units (264 municipalities and 480 rural municipalities) [22].

We used a random sampling technique with multiple stages. At first stage, within the Kaski district, out of 4 rural municipalities and 1 urban municipality, 1 ward was randomly selected from each rural municipality and 4 wards from urban municipality to maintain the proportion, using the lottery method. In this method, we assigned a specific number to each ward of 4 rural municipalities and 1 urban municipality and wrote all the numbers on separate pieces of paper. These pieces of paper were mixed and put into two boxes each for rural and urban municipalities, from which numbers were drawn out in a random manner [23]. The randomly selected wards for the rural municipality were ward no. 2 (Rupa), ward no. 4 (Madi), ward no. 6 (Annapurna) and ward 4 (Machapuchhre). Similarly, the selected wards for the urban municipality were ward 5, 11, 20 and 32.

Next, we selected the participants randomly from each ward using spin the bottle method. In this method, we went to the center of the ward and span the bottle. We then proceeded towards the direction the bottleneck indicated, and selected household until the desired sample in that ward was achieved. If the required sample was not fulfilled from the projected direction, then the technique was repeated at the same junction and the households were selected in the same manner in the other direction to fulfill the desired sample of the respective ward. We selected participants from each household on the basis of their availability during the data collection and inclusion criteria [24].

Nepalese adults aged 18 years and above residing in the respective wards of Kaski district and giving their consent to participate were the study population. Participants who were diagnosed with some kind of cancer and who have had a history of cancer were excluded from the study.

### Sample size

We calculated the sample size at the level of 95% significance level, with prevalence (p) as 50%, permissible error of 5% and non-response rate of 10% [25]. We chose 50% prevalence of cancer screening behaviour since there was no relevant prevalence available in literatures for this particular study in this particular setting. We used the Cochran’s Sample Size formula for an unknown population (z^2^pq/e^2^) and the calculated sample size was 188 [26]. To draw proportionate sample from each ward (4 rural and 4 urban), we decided to select 24 participants from each ward, and hence, approached 192 participants. However, we had response rate of 93.8%, i.e. 180 participants.

### Study variables and instruments

A semi-structured questionnaire was developed incorporating socio-demographic variables, need factors, pre-disposing factors, enabling factors, cancer literacy and cancer screening behavior.

Cancer has been operationalized as a broad category of all cancer types in this study for the feasibility during data collection. Socio-demographic variables noted were age, gender, residence, marital status, education level, ethnicity, religion, occupation and family income. Residence was classified as rural and urban; marital status as ‘married’ and ‘others’ (includes unmarried and widowed); education level as ‘no formal education’ (includes illiterate) and ‘formal education’ (Grade 1 through tertiary level of education); ethnicity as ‘upper caste groups’ and ‘others’ (according to classification by Ministry of Health and Population [27]); religion as ‘Hindu’ and ‘others’ (Buddhist and Christian); occupation as homemaker, business and others (agriculture, private and government jobs, student); and family income in comparison to median income of NRs. 30,000.

Need, pre-disposing and enabling factors were based on Andersen’s framework [28] and conceptual framework by Lee et al. [18]. The need factors in this study were family history of cancer, history of other chronic disease and self-rated health status; pre-disposing factors were use of naturopathy and other home remedies and fatalism; and enabling factor was family support.

We calculated the scores for the item related to the use of naturopathy and other home remedies, fatalism and family support as per the study by Lee et al. [18]

Use of other medicines to treat disease included 5 point Likert scale items—"I sometimes use home remedies or naturopathy to cure the illness before trying western medicine or allopathy" and use of other medicines to treat disease—"I believe naturopathy/home medicine is very effective in treating health problems" [18].

Similarly, for the fatalism, items included were "It seems like almost everything causes cancer", "There is not much people can do to lower their chances of getting cancer" and “There are so many recommendations about preventing cancer, so it is hard to know which ones to follow" [18].

Finally, for family support, the items included were—"My adult children or other family relatives have recommended me to get checked for cancer", "My family or other family relatives have talked to me about the importance of getting checked for cancer" and "I rely on my family to advise me about health matters” [18].

For cancer literacy, we utilized 6-item Cancer Health Literacy Test (CHLT-6) to assess cancer literacy score [19]. Since there are 6 questions in the tool, the total possible score for each participant was 6.

For cancer screening behavior, we classified the screening behaviour on the basis of self-reported information regarding screening for cancer about whether they had ever got screened for any type of cancer.

The content validity of the research instrument was established by seeking opinions from the experts and research investigators. We pre-tested the tool on a similar sample population (of Kathmandu and Bhaktapur districts) of 30 participants to check the accuracy of the content [29]. The internal consistency (reliability) was measured via Cronbach’s alpha, which was found to be 0.722. We made necessary changes, such as wordings and sequence of the questions, modification in options and addition/removal of more/less relevant questions as per the suggestions and further literature review.

### Data collection

Data collection was carried out with the aid of face-to-face interviews by door to door visits to systematically selected random households by the researchers.

### Data management and analysis

We entered, cleaned and coded data in Microsoft Excel 2011 and then exported to Statistical Package for Social Science (SPSS) software, version 11.5, for statistical analysis [30]. Alphanumeric codes were used. For monitoring, data was entered on a regular basis.

Frequencies and percentages were calculated to identify the distribution of socio-demographic information. A Shapiro’s Wilk test and a visual inspection of their histograms, normal Q-Q plots and box plots showed that the variables such age, income, use of naturopathy and other home remedies, fatalism, family support and cancer literacy scores were not approximately normally distributed for both categories of those who have and have not gone through any cancer screening test in the past. Hence, their median scores and inter-quartile range have been reported. The multivariate logistic regression model was derived to find the relationship among the variables. The odds ratio was calculated to calculate the strength of associations among variables.

All statistical tests were two-tailed, and associations were considered to be statistically significant for a p-value less than 0.05 tested at 95% Confidence Interval.

## Results

### Socio-demographic characteristics of the study participants

The total sample studied was 180, of which 63.8% were females and 36.1% males. “Table 1” shows the socio-demographic characteristics of the study participants on the basis of the outcome variable (cancer screening). The median age (with IQR) of the participants who had gone through some kind of cancer screening test was 42.0 (34.5–58.5) years. Majority of them were married (81%), had formal education (80.6%), and followed Hinduism (80.0%). There were more in Upper caste groups who had gone through any kind of cancer screening test in the past.

**Table 1:** Socio-demographic characteristics of the study participants (n = 180).

Characteristics	Categories	Cancer Screening	
		Ever Screened (n = 21)	Never Screened (n = 159)
		n (%)	n (%)
**Age** in years (Median, IQR)		42 (34.5–58.5)	33 (26–45)
**Age groups**	18–25	0	38 (100.0)
	26–35	6 (9.7)	56 (90.3)
	36–45	7 (20.6)	27 (79.4)
	46–55	3 (23.1)	10 (76.9)
	56–65	2 (9.5)	19 (90.5)
	> 65	3 (25.0)	9 (75.0)
**Gender**	Male	2 (3.1)	63 (96.9)
	Female	19 (16.5)	96 (83.5)
**Residence**	Rural	12 (13.2)	79 (86.8)
	Urban	9 (10.1)	80 (89.9)
**Marital Status**	Married	20 (13.7)	126 (86.3)
	Others	1 (2.9)	33 (97.1)
**Education**	No formal education	4 (11.4)	31 (88.6)
	Formal education	17 (11.7)	128 (88.3)
**Ethnicity**	Upper caste groups	11 (14.5)	65 (85.5)
	Other caste groups	10 (9.6)	94 (90.4)
**Religion**	Hindu	18 (12.5)	126 (87.5)
	Others	3 (8.30	33 (91.7)
**Occupation**	Homemaker	6 (12.0)	44 (88.0)
	Business	8 (11.6)	61 (88.4)
	Others	7 (11.5)	54 (88.5)
**Family’s Monthly Income** (NRs.) (Median, IQR)		30,000 (20,000–50,000)	30,000 (20,000–50,000)

### Need, pre-disposing and enabling factors of cancer screening behaviour among the study participants

“Table 2” shows the need, pre-disposing and enabling factors of cancer screening behaviour among the study participants.

**Table 2:** Need, pre-disposing and enabling factors of cancer screening behaviour among the study participants (n = 180).

Characteristics	Categories	Cancer Screening	
		Ever Screened (n = 21)	Never Screened (n = 159)
		n (%)	n (%)
**Need factors**	**Family history of cancer**		
	Yes	6 (20.0)	24 (80.0)
	No	15 (10.0)	135 (90.0)
	**History of other chronic disease**		
	Yes	5 (15.6)	27 (84.4)
	No	16 (10.8)	132 (89.2)
	**Self-rated health status**		
	Good	20 (11.8)	150 (88.2)
	Bad	1 (10.0	9 (90.0)
**Pre-disposing factors**	**Use of naturopathy and other home remedies**		
	Median [IQR]	0.0 [(-2.0)– 1.0]	0.0 [(-2.0)– 1.0]
	**Fatalism**		
	Median [IQR]	-1.0 [(-1.5)– 1.0]	0.0 [(-1.0)– 1.0]
**Enabling factor**	**Family support**		
	Median [IQR]	3.0 [(-1.0)– 3.5]	-1.0 [(-1.0)– 1.0]

Majority of the participants who had not got screened for cancer (90.0%) reported no history of cancer in their families. Similarly, many of those denying getting screened for cancer, did not have history of other chronic disease (89.2%), and reported their health status to be good (88.2%).

As for the pre-disposing and enabling factors, the median scores (with IQR) for use of naturopathy and other home remedies, fatalism and family support among those who reported getting screened for cancer in the past, were found to be 0.0 [(-2.0)– 1.0], -1.0 [(-1.5)– 1.0] and 3.0 [(-1.0)– 3.5] respectively.

### Cancer literacy among the study participants

As for the cancer literacy score, the participants were asked six questions of Cancer Health Literacy Test (CHLT-6) and for each correct response, the participant received one score each. The median cancer literacy score for those who had gone through cancer screening test was 4 with interquartile range of 2 to 5.5. Similarly, the median score for those who had not taken any cancer screening test was 3 with interquartile range of 2 to 4. Here, the median score for the former is slightly higher than that of the latter. “Table 3” shows the number of participants receiving certain cancer literacy score. Less than half (43.4%) of the participants scored more than median score (3) for cancer literacy.

**Table 3:** Cancer literacy scores among the study participants (n = 180).

Characteristics	Categories	Frequency	Percentage
**Cancer Literacy Score**	0	19	10.6
	1	18	10.0
	2	33	18.3
	3	32	17.8
	4	36	20.0
	5	23	12.8
	6	19	10.6

### Cancer screening behaviour among the study participants

As for the cancer screening behavior, only 11.7% of the respondents had ever undergone cancer screening test, out of which only 33.3% had gone through it in last six months. Almost two-thirds (64.3%) had taken a screening test for cervical cancer followed by breast cancer (21.4%), prostate cancer (3.6%) and throat cancer (3.6%) (“Table 4”).

**Table 4:** Cancer screening behaviour among the study participants (n = 180).

Characteristics	Categories	Frequency (n)	Percent (%)
**Ever done cancer screening?**	Yes	21	11.7
	No	159	88.3
If Yes, for which cancer type?*	Cervical	18	85.7
	Breast	6	28.6
	Prostrate	1	4.8
	Throat	1	4.8
	Don’t know the name	2	9.5
**If Yes, when did you do the screening?**	≤ 6 months ago	7	33.3
	> 6 months ago	14	66.7

* Multiple response question

### Factors associated with cancer screening behaviour among the study participants

“Table 5” shows simple logistic regression analysis of socio-demographic characteristics; need, pre-disposing and enabling factors; and cancer literacy scores with cancer screening behaviour. Age, gender and family support were found to be significantly associated with cancer screening behaviour with p-value less than 0.05 in this model. On the other hand, no significant association was found in other factors like residence, marital status, education, ethnicity, religion, occupation, family’s monthly income, family history of cancer, history of other chronic disease, self-rated health status, use of naturopathy and other home remedies, fatalism and cancer literacy.

**Table 5:** Simple logistic regression analysis of socio-demographic characteristics, need, pre-disposing and enabling factors; and cancer literacy scores with cancer screening behaviour among the study participants (n = 180).

Characteristics	Categories	COR (95% CI)	p—value
Age		1.03 (1.00–1.06)	0.025*
Gender	Male	0.16 (0.04–0.71)	0.016*
	Female	Ref	
Residence	Urban	0.74 (0.30–1.87)	0.522
	Rural	Ref	
Marital Status	Married	5.24 (0.68–40.47)	0.112
	Others	Ref	
Education	No formal education	0.97 (0.31–3.09)	0.961
	Formal education	Ref	
Ethnicity	Upper caste groups	1.59 (0.64–3.97)	0.319
	Other caste groups	Ref	
Religion	Hindu	1.57 (0.44–5.66)	0.489
	Others	Ref	
Occupation	Homemaker	1.05 (0.33–3.36)	0.932
	Business	1.01 (0.34–2.98)	0.983
	Others	Ref	
Family history of cancer	No	0.62 (0.24–1.60)	0.325
	Yes	Ref	
History of other chronic disease	No	0.66 (0.22–1.94)	0.444
	Yes	Ref	
Self-rated health status	Bad	0.83 (0.10–6.93)	0.866
	Good	Ref	
Use of naturopathy and other home remedies		1.00 (0.79–1.25)	0.966
Fatalism		0.90 (0.71–1.13)	0.360
Family support		1.43 (1.15–1.77)	0.001*
Cancer literacy		1.24 (0.95–1.63)	0.109

* Significant at p-value < 0.05

Then, the variables with p-value less than 0.2 were entered onto a multivariate logistic regression model. Here, age (OR = 1.06, C.I. = 1.02–1.11), gender (OR = 0.06, C.I. = 0.01–0.35) and cancer literacy (OR = 1.43, C.I. = 1.01–2.02) turned out to be significant predictors of cancer screening behaviour. In other words, females, those of older age and those with higher cancer literacy scores were more likely to get screened for cancer (“Table 6”).

**Table 6:** Multivariate logistic regression analysis of socio-demographic characteristics; need, pre-disposing and enabling factors; and cancer literacy scores with cancer screening behaviour among the study participants (n = 180).

Characteristics	Categories	AOR (95% CI)	p—value
**Age**		1.06 (1.02–1.11)	0.005*
**Gender**	Female	Ref	0.002*
	Male	0.06 (0.01–0.35)	
**Marital Status**	Others	Ref	0.227
	Married	3.74 (0.44–31.72)	
**Family support**		1.25 (0.96–1.63)	0.093
**Cancer literacy**		1.42 (1.01–2.02)	0.045*

* Significant at p-value < 0.05

## Discussion

Findings from 180 adults from Kaski district of Nepal, showed that only 11.7% of the participants had ever screened for any cancer in the past. Older age, female gender and higher cancer literacy scores were associated with cancer screening behaviour. Less than half (43.4%) of the participants scored more than median score for cancer literacy.

The proportion of study participants reporting to have ever screened for cancer is similar to researches done in Nepal and other low-income countries, ranging from 2.4% to 18.3% [7–10, 16, 17, 31, 32]. The lower proportion may be due to the less focus on cancer screening programs in comparison to communicable diseases in the country, and also general nature of the public to not go to health centers and clinics for preventive care and only visiting these facilities for treatments and other care [4, 33].

Our study showed only 43.4% of the respondents scored more than the median score cancer literacy. This was somehow different from the study done in United States, which had 82% of respondents with adequate cancer literacy, i.e. with higher cancer literacy scores [19]. This difference can be mainly due to the fact that the latter study was done among distinctive population who are known to have higher literacy rates as well as the population comprised of all cancer patients, who might be exposed to cancer related terminologies in some way or the other during the process of diagnosis and treatment.

Cancer literacy was found to be a significant factor in determining the tendency for cancer screening among adults in this study, like one done in South Korea where cancer literacy had a mediating role in the relationship between population characteristics and cancer screening behaviors [18]. Similarly, the participants with adequate Cancer Literacy were found to be five times more likely to have been screened for cancer than others in a study conducted in Switzerland [34]. In our study, the participants with higher cancer literacy scores were 1.43 times more likely than the others to have been screened for cancer. Nevertheless, we believe more comprehensive measurement, such as Cancer Health Literacy Test– 30 (CHLT-30) for cancer literacy can be contextualized and utilized which may give more perspective to cancer literacy [19]. Further qualitative and quantitative studies may provide more insight to this measure, as this was presumably the first study to assess cancer literacy in this region.

There was a higher frequency of women in the higher age groups going through screenings in comparison to the lower age groups in our study. This finding was in line with the studies carried out in central Nepal, Nigeria, Turkey and Kenya, however, age was not a significant factor for cervical cancer screening uptake in these studies [17, 31, 35–37]. In a country like Nepal where people go to hospitals only when needed, the older tend to visit hospitals and clinics more than the younger ones, which might lead to more screening incidence [33]. Also, the cultural and social knits of Nepalese society where respecting and caring for elders is a prevailing value, specially in rural areas, may have some role in older age groups being taken care of more than the younger ones [38].

In addition, gender was significantly associated with cancer screening. Cancer screening camps targeting women, which are feasible in the community setting such as for Cervical and Breast cancers are organized frequently in Nepal and this might have led more cancer screening practices among females than males [39]. This finding was similar to the studies conducted in South Korea and among South Asian immigrants in the UK, US and Canada where gender is shown to be a significant predictor of cancer screening behaviour [18, 40]. However, it was different than that of researches carried out in US and Switzerland [19, 34]. There was preponderance of female respondents with proportion of 63.9% in this study. This was mainly due to the fact that there were more female homemakers present during the time of data collection. The households did not have presence of male counterparts at that time who might be at formal or informal work settings outside their houses, and hence could not participate in this study.

Yet, history of cancer in immediate family members was not found significant after adjusting the confounders in this study, which is different from the researches done in central Nepal and South Korea which found that family history of cancer can determine cancer screening behaviour among the respondents [8, 18]. This may be due to various factors, ranging from socio-economic status to general human behaviour. Cancer treatment is costly, and this can be the huge factor for people with family members not opting for regular cancer screening because they might think being ignorant about it better than learning one has cancer and going through the optimal financial and mental stress. More qualitative studies are required to go into depth of this health-related behaviour of utilization of health resources. Nevertheless, the study conducted in South Korea has consistent results regarding family history of cancer with this study [18].

Residence, education, self-rated health status and fatalism did not have significant association with cancer screening behavior in this study which is different than many other studies carried out in South Korea, some low income countries and other parts of Nepal [8, 9, 16, 18, 31, 32].

The strengths of this study are random sampling, coverage of both rural and urban areas, adding evidence to researches on non-communicable diseases in a country like Nepal where there is more focus on communicable diseases, and exploring a relatively new arena of understanding of cancer prevention and management. We acknowledge that the study also had a number of limitations. Firstly, our study was based on the self-reported information provided by the participants, which may have led to some information bias. Secondly, our study could not examine the causal relationship due to the nature of its study design, for which further longitudinal studies looking at cancer screening behaviour may be needed. Lastly, as this study was carried out in, though randomly, selected municipalities of a particular hilly district, the findings may not be generalizable to other mountainous and plain regions of the country, where level of awareness about cancer as well as access to health facilities may differ from this region. Nevertheless, this study has demonstrated the need to expand cancer screening services to people and come up with ideas for them to opt it on a regular basis, so that the rising burden of cancer in Nepal can be minimized.

## Conclusion

This study presented low percentage of the respondents had ever gone through screening for cancer of varying types. The factors such as age, gender and cancer literacy were found to be significant predictors to determine cancer screening behaviour among the study participants. These prevalent findings may suggest a possible increase in the burden of non-communicable diseases like cancer in a low-income country like Nepal.

Our study recommends opportunistic cancer screening services to boost screening rate for those who come for other general examinations. Education and awareness campaigns can be conducted to enhance cancer literacy.

## Supporting information

S1 File (XLS)
